# Wnt Signaling Cascades and Their Role in Coronary Artery Health and Disease

**DOI:** 10.33696/Signaling.2.035

**Published:** 2021

**Authors:** Nadisha Weerackoon, Kushan L. Gunawardhana, Arya Mani

**Affiliations:** 1 Section of Cardiovascular Medicine, Department of Internal Medicine, Yale University School of Medicine, New Haven, CT 06510, USA; 2 Department of Genetics, Yale University School of Medicine, New Haven, CT 06510, USA

**Keywords:** Wnt signaling, β-catenin, Cardiovascular disease, Atherosclerosis, VSMCs, Endothelial dysfunction, Hyperlipidemia, Metabolic syndrome, Diabetes, Myocardial infarction

## Abstract

The Wnt signaling is classified as two distinct pathways of canonical Wnt/β-catenin signaling, and the non-canonical pathways of planar cell polarity and Wnt/Ca^2+^ pathways. However, the scientific discoveries in recent years have shown that canonical and non-canonical Wnts pathways are intertwined and have complex interaction with other major signaling pathways such as hedgehog, Hippo and TOR signaling. Wnt signaling plays important roles in cell proliferation, differentiation and migration during embryonic development. The impairment of these pathways during embryonic development often leads to major congenital defects. In adult organisms Wnt expression is more restricted to proliferating tissues, where it plays a key role in tissue regeneration. In addition, the disruption of homeostatic processes of multicellular organisms may give rise to reactivation and/or altered activation of Wnt signaling, leading to development of malignant tumors and chronic diseases such as type-2 diabetes and adult cardiovascular diseases.

Coronary artery disease (CAD) is the leading cause of death in the world. The disease is the consequences of two distinct disease processes: Atherosclerosis, a primarily inflammatory disease and plaque erosion, a disease process associated with endothelial cell defect and smooth muscle proliferation with only modest contribution of inflammatory cells. The atherosclerosis is itself a multifactorial disease that is initiated by lipid deposition and endothelial dysfunction, triggering vascular inflammation via recruitment and aggregation of monocytes and their transformation to foam cell by the uptake of modified low-density lipoprotein (LDL), culminating in an atheromatous plaque core formation. Further accumulation of lipids, infiltration and proliferation of vascular smooth muscle cells (VSMCs) and extracellular matrix deposition result in intimal hyperplasia. Myocardial infarction is the ultimate consequence of these processes and is caused by plaque rupture and hypercoagulation. *In vivo* studies have established the role of the Wnt pathway in all phases of atherosclerosis development, though much remains unknown or controversial. Less is known about the mechanisms that induce plaque erosion. The limited evidence in mouse models of Wnt coreceptor LRP6 mutation and heterozygous TCF7L2 knock out mice implicate altered Wnt signaling also in the pathogenesis of plaque erosion. In this article we focus and review the role of the Wnt pathway in CAD pathophysiology from clinical and experimental standpoints.

## Introduction

More than 30 years ago very first Wnt proteins were discovered as members of a family of proteins involved in the development of multicellular organisms [1,2]. The human genome contains 19 Wnt proteins which are post-translationally modified via palmitoylation and glycosylation in the endoplasmic reticulum by porcupine [3,4], a crucial step in their secretion and receptor binding. Beside their multipotent role during development, Wnt proteins play a vital role in adult tissue homeostasis and regeneration [5]. In adult tissues, cell proliferation and differentiation and stem cell renewal are largely controlled by the secreted Wnt proteins. While numerous Wnt-based signaling pathways have been identified, the Wnt-β-catenin pathway is the most explored pathway and thus referred to as the ‘canonical’ Wnt pathway. It is an evolutionarily conserved cell-cell communication pathway [3] that guides stem cell renewal, proliferation and differentiation via β-catenin co-transcriptional activities [6]. Historically, the Frizzled (FZD) and LRP5/6 molecules were the first proteins implicated as receptors for Wnt ligands [7]. FZD proteins consist of a seven-pass transmembrane portion and an extracellular cysteine-rich domain (CRD) [8]. The secreted Wnt proteins function by simultaneous binding of FZD-LRP5/6 receptors on nearby cells, leading to serine and threonine phosphorylation of the LRP5/6 tail within PPPSP motif. Wnt proteins bind with high affinity to the CRD of multiple FZDs, while a single FZDs can also interact with multiple Wnts [9,10]. In humans, 10 FZD protein homologs exist that contain 120 amino acid extracellular CRDs for Wnt binding. Several other discoveries of the signaling pathway components, such as its endogenous antagonists have deemed it intricate yet fascinating pathway [2].

## Wnt Cascade Mechanism, Activators, Inhibitors

**Figure 1:**
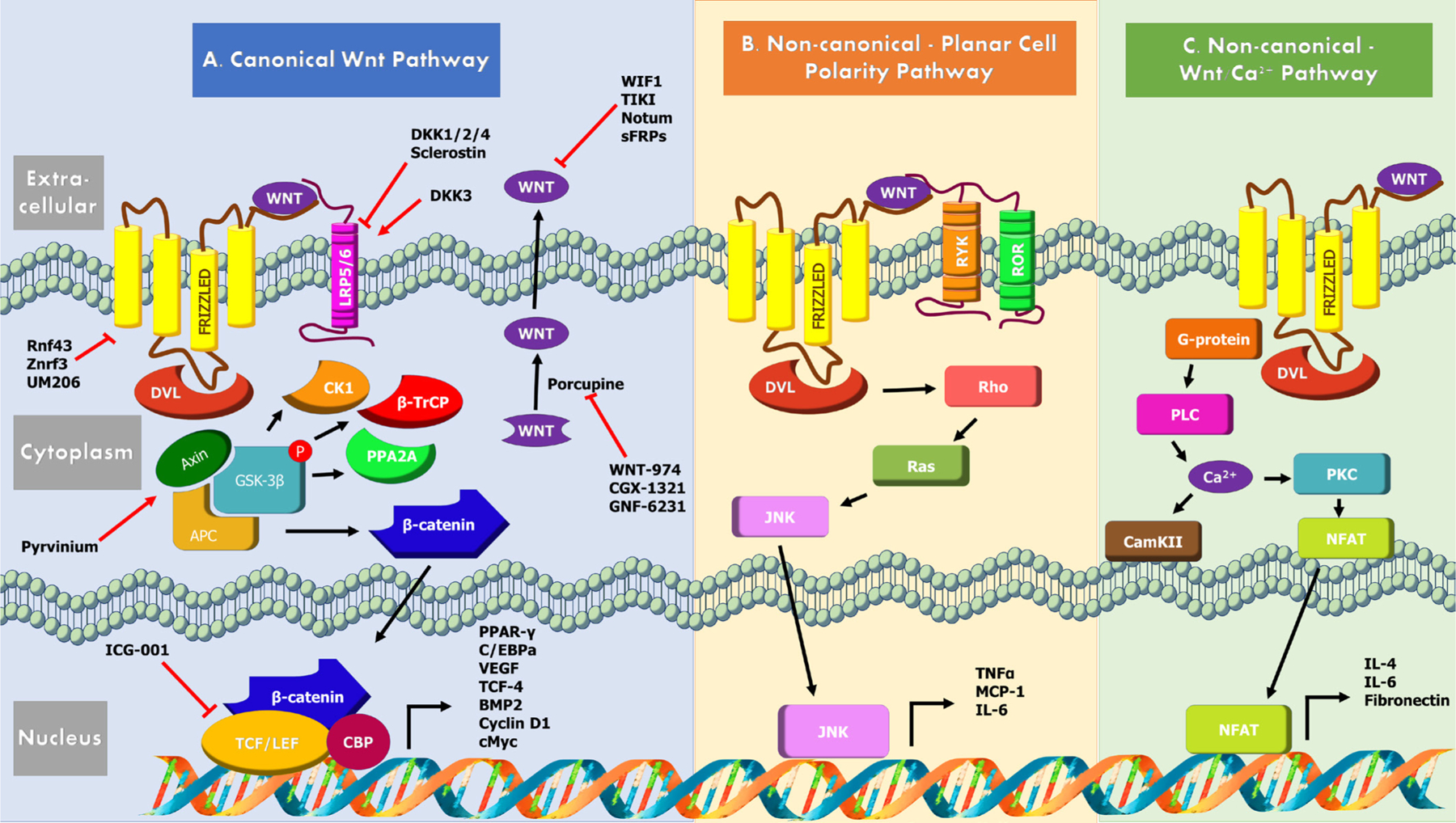
Overview of canonical and non-canonical Wnt signaling pathways. **(A)** In *canonical Wnt signaling*, binding of Wnt ligands to Fzd receptors and LRP co-receptors activates the canonical pathway. CK1α and GSK3β phosphorylate LRP receptors which activate DVL. β-catenin is phosphorylated in the absence of Wnt ligands by the protein destruction complex comprising of Axin, APC and GSK3β, CK1α, PP2A and β-TrCP. The destruction complex is inactivated by DVL and β-catenin accumulation and translocation to the nucleus occurs. In the nucleus, β-catenin forms a complex with LEF and TCF. Downstream target genes PPAR-γ, CCAAT/(C/EBPα), VEGF, TCF-4 and BMP2 are transcribed via the TCF/LEF promoter, due to increased β-catenin. **(B)** In *non-canonical Wnt signaling*, Wnt binds to Fzd receptors and RYK/ROR coreceptors to activate DVL. DVL binds and activates GTPase Rho ad Ras-related C3 Botulinum toxin substrate and JNK which regulate downstream genes via AP-1 including TNF-α, MCP-1, and IL-6. **(C)** The *Wnt/Ca*^*2+*^ pathway involves G-protein and PLC activation via Wnt signals with FZD in the dearth of LRP5/6. Increased intracellular Ca^2+^ occurs and PKC is activated. Downstream target genes (including IL-6, IL-4 and fibronectin) are activated via T cell promoter. Due to Ca^2+^dependent calmodulin kinase activation β-catenin activation is suppressed. Frizzled (Fzd); Dishevelled (DVL); low-density lipoprotein receptor 5/6 (LRP5/6); adenomatous polyposis coli (APC); glycogen synthase kinase (GSK)-3β; protein phosphatase 2A (PP2A); Casein Kinase 1 (CK1); Beta transducin repeats-containing protein (β-TrCP); T cell factor/lymphocyte enhancer factor (TCF/LEF); Dickkopf (Dkk); Peroxisome proliferator-activated receptor gamma (PPAR-γ); CCAAT-enhancer-binding protein alpha (C/EBPα); vascular endothelial growth factor (VEGF); Bone morphogenic protein 2 (BMP2); creb binding protein (CBP); receptor tyrosine kinase (RYK); receptor tyrosine kinase-like orphan receptor (ROR); Ras-related C3 Botulinum toxin substrate (Ras); Wnt/Jun N-terminal kinase (JNK), Tumor necrosis factor alpha (TNF-α); monocyte chemotactic protein-1 (MCP-1); Interleukin-6 (IL-6); protein kinase C (PKC); phospholipase C (PLC); Ca^2+^/calmodulin-dependent protein kinase (CAMKII); nuclear factor of activated T-cells (NFAT).

Discovered initially as Int-1, Wnt was subsequently found to be a homologous of wingless gene in Drosophila [11]. The Wnt signaling pathway comprises of the Wnt protein family, FZD receptors, co-receptors LRP5/6, disheveld (DVL), adenomatous polyposis coli (APC), cytoplasmic protein β-catenin, glycogen synthase kinase (GSK)-3β, axin and T cell factor/lymphocyte enhancer factor (TCF/LEF) transcription factors (Figure 1).

The Wnt signaling starts by the release of Wnt proteins into the extracellular space by GPR177 (the vertebrate homolog of Drosophila Wingless/Evi/Sprinter), an escort protein that utilizes endosome trafficking of Wnt to the plasma membrane. A major factor for Wnt binding is the Wnt palmitoleic acid moiety which presents itself in the CRD within a hydrophobic fissure.

The canonical Wnt-β-catenin pathway is involved in post-translational control of β-catenin. β-catenin is a chief downstream effector of the canonical Wnt signaling, which is degraded in in the absence of a Wnt stimulus by a multiprotein “destruction complex” that includes Axin, APC, GSK-3β, Casein kinase 1 (CK1), protein phosphatase 2A (PP2A), and the E3-ubiquitin ligase β-TrCP [6]. The complex generates a β-TrCP recognition site by phosphorylation of a conserved Ser/Thr-rich sequence near the β-catenin amino terminal and its ubiquitin-dependent proteosomal degradation ensures reduced cytoplasmic β-catenin levels via the β-catenin destruction complex. When Wnt is present in the extracellular space, the canonical Wnt signaling is initiated by the simultaneous binding of Wnt secreted glycoproteins to N-terminal extracellular CRD of the seven-transmembrane-span heterodimeric FZD receptor and its single transmembrane domain containing co-receptors LRP5/6. This ligand-receptor interaction triggers confirmation changes in the receptors permitting FZD interacting with Dvl through the PDZ domain, which subsequently results in its recruitment by destruction complex via its DIX domain [6]. LRP6 is phosphorylated by GSK3 β, priming its further phosphorylation by Ck1. Formation of a complex between GSK3 β, APC and axin prevents cytoplasmic phosphorylation of β-catenin and its degradation. β-catenin then enters the nucleus and transcribe downstream target genes in combinatorial fashion with TCF/LEF family of transcription factors. Some of the known targets of Wnt/β-catenin include Peroxisome proliferator-activated receptor gamma (PPAR-γ), CCAAT-enhancer-binding protein alpha (C/EBPα), vascular endothelial growth factor (VEGF), TCF-4 and Bone morphogenic protein 2 (BMP2) [6].

Non-canonical pathways, are β-catenin independent, regulate cell polarity and cell movement via Rho GTPases or control calcium (Ca^2+^) signaling via G proteins [3]. The non-canonical Wnt/JNK pathway is activated when Wnt binds to cell surface FZD family of G-coupled protein receptors and derailed receptor tyrosine kinase (RYK) or receptor tyrosine kinase-like orphan receptor (ROR), leading to activation of downstream GTPase Rho and Ras-related C3 botulinum toxin substrate and JNK which regulate cytoskeleton activities [12]. The Wnt/Ca^2+^ pathway involves activation of phospholipase C, short-lived increase in the concentration of certain intracellular signaling molecules, inositol 1,4,5-triphosphate (IP3), 1,2 diacylglycerol (DAG) [13]. IP3 diffuses through the cytosol and interacts with the calcium channels on the endoplasmic reticulum triggering the release of calcium into cytoplasm and subsequent activation of the calcium calmodulin-dependent protein kinase II (CaMKII), calcium calcineurin and Nuclear factor of activated T-cells (NFAT) axis. DAG and released calcium together activate protein kinase C (PKC). In T cells this causes transportation of NFAT to nucleus and activation of downstream target genes (namely IL-6, IL-4 and fibronectin). NFAT also directly interacts with DVL, in a Ca^2+^ dependent manner and inhibits canonical Wnt/ β-catenin signaling pathway [14–16].

Inhibition of Wnt signaling occurs at multiple levels. Tiki and Notum are extracellular enzymes that cleave N-terminus of Wnts or detach their palmitoleate moiety [17]. Direct inhibition of Wnts occur via secreted proteins such as the Wnt Inhibitory Factor (WIF) or secreted FZD related proteins (sFRPs) that prevent Wnt interaction with receptor complexes [18]. Dkk and sclerostin are Wnt signaling inhibitors that bind to LRP receptors to antagonize function. Dkk1, Dkk2 and Dkk4 are LRP5/LRP6 ligands which antagonize Wnt and canonical pathways. In contrast, Dkk3 activates canonical pathways [19]. Dkk4 also indirectly activates c-Jun non-canonical pathway. Sclerostin binds LRP4, LRP5 and LRP6, but unlike Dkk, is not a competitive inhibitor of Wnt [20]. Wnt/β-catenin target genes Ring finger protein 43 (Rnf43) and Zinc and Ring Finger 3 (Znrf3) are E3 ubiquitin-protein ligases that negatively regulate Wnt signaling by reducing Wnt receptors FZD and LRP6 at the plasma membrane via endocytosis and degradation [21]. They are both neutralized by Lgr5/R-spondin complex.

At the cytoplasmic level, Axin2 is involved in a negative feedback loop by the formation destruction complex and β-catenin degradation. Naked cuticle 1 (*NKD1*) is yet another Wnt target gene that promotes negative feedback via either DVL inhibition or prevention of β-catenin nuclear import [22]. Lastly, inhibitor of β-catenin and T-cell factor (ICAT), an 81 amino acid protein directly inhibits β-catenin in the nucleus thus inhibiting Wnt signaling in the nervous system [3].

## Cardiovascular Development, Coronary Artery Disease and Wnt Signaling

At the gastrulation stage Wnt is necessary for the maintenance of undifferentiated cardiac progenitor cells whereas at the post-gastrulation stages Wnt inhibition stimulates differentiation of cardiomyocytes [11]. The β-catenin and non-β-catenin-mediated Wnt signaling pathways are vital to the generation of cardiac progenitor cells [23]. Accordingly, mice that are devoid of β-catenin lack mesoderm. This suggests that β-catenin signaling is a requirement for the formation of the mesoderm, a source of cardiac progenitor cells. A key player that facilitates accurate heart development is Wnt11, which uses the non-β-catenin-mediated signaling pathway. Cardiac neural crest cell migration and differentiation and formation of outflow tract and valve formation are dependent on the Wnt signaling pathways [11]. The conduction system development is another cardiac component controlled by the Wnt signaling. Although Wnt7a and Wnt11 have been speculated to be upregulated during the formation of the conduction system in chicken embryo, no functional data exists to support their direct role in the process [24].

The perturbation of Wnt signaling plays a significant role in the development of adult cardiac diseases, including atherosclerosis. The first evidence linking Wnt signaling to atherosclerosis came from a discovery of a single gene mutation in the LRP6 gene in a multiplex family with early onset CAD and myocardial infarction, type 2 diabetes, hyperlipidemia and hypertension [25] and later in several nuclear families. Another study by the same group showed that that the mice with the human LRP6^R611C^ mutation developed modest dyslipidemia but advanced proliferative CAD, which could be reversed by enhancing canonical Wnt signals in LRP6 with Wnt3a injections [25]. The Wnt coreceptor Lrp5 has been shown to play an important for macrophage phagocytosis and clearance of LDL as shown in Lrp5^−/−^ mice, which develop atherosclerosis on a high fat diet [26]. The characterization of LRP5 has shown that it inhibits infiltration of aortic macrophages and cytokine release [20]. Wnt5 has been shown to upregulate Abca1 in ox-LDL treated RAW264.7 cells and reduce lipid accumulation by upregulating reverse cholesterol transport [27]. Accordingly, the plasma levels of DKK1 have been found to be high in patients with atherosclerosis. This has been attributed to increased expression and release of DKK1 from platelets [2]. Further, the circulating levels of Wnt antagonist Dkk1 has been shown to be higher in acute ischemic stroke and symptomatic aortic stenosis patients [20]. A common variant of LRP6 (*p.1062V*) was found to be strongly associated with carotid artery atherosclerosis. Whether the risk allele results in reduced or increased LRP6 expression is unclear at this point [28].

While the aforementioned studies have shown that impaired function of Wnt signaling components contributes to atherosclerosis, there are other studies that have shown deleterious effects of canonical Wnt pathway on cardiovascular system. The increased pro-proliferative effect of β-catenin on VSMCs via cyclin D1 activation and reduced cell cycle inhibition has been shown by several studies [29]. Wnt1 and Wnt3A both have been shown to provoke cyclin D1 expression in VSMCs [30]. Wnt4 and Wnt5A have also been seen to be increased in injured carotid arteries [2]. The role of Wnt5A in the disease process is, however, complicated as in the presence of FZD4 it activates beta-catenin signaling while in the presence of ROR2, it inhibits the canonical Wnt pathway [31]. Several Wnt inhibitors have been identified that may alter cardiovascular disease outcome. Sfrp5, an adipocytokinine secreted by the adipose tissue, acts as an inhibitor of Wnt function. SFRPs share a similar CRD to that of FZD. Thereby, it can inhibit Wnt signaling through binding to FZD and forming non-functional complexes [14]. In mice, Sfrp5 has been shown to prevent myocardial inflammation and injury *in vivo*, as it inhibits Wnt5a activation of JNK and expression of inflammatory genes [32]. Sfrp2, another member of the Sfrp family, supports myocardial stem cell survival and repair via influencing cell polarity that diminishes fibrosis in mice [33,34].

Although many molecules that target Wnt pathway have been identified, only few have been shown to be effective and specific. Among these is GNF-6231, an inhibitor of porcupine that has been shown to reduce myocardial injury and rescue myocytes after myocardial infarction [35]. This therapeutic inhibition of Wnt signaling has positively impacted multiple aspects of infarct recovery; it promotes cell proliferation of cardiac progenitors and interstitial cells, improves cardiomyocyte survival, inhibit myofibroblast proliferation and reduce collagen type-1 proteins in them. Another molecule is tankyrase, an antagonist of the poly ADP-ribose polymerase, which reduces Axin levels and lead to diminished Wnt signaling. Treatment with this molecule has shown to reduce mechanical injury-induced neointima formation, evident by the loss of intima area and attenuated proliferation, migration and cell cycle arrest of VSMCs [20,36].

## Endothelial Dysfunction and Wnt

Increased Wnt/β-catenin activation may induce endothelial dysfunction (ED). Wnt5a has been shown to induce cyclooxygenase-2 expression and enhance inflammatory cytokines such as IL-8 in human aortic ECs, likely via noncanonical Wnt/Ca^2+^/protein kinase C pathway [37]. Studies in human coronary artery endothelial cells (HCAEC) have shown that Wnt5A may also enhance permeability through Ryk interaction and downstream ROCK/LIMK2/CFL1 signaling [38]. The canonical Wnt pathway may also induce vascular endothelial dysfunction via redox regulatory protein p66(Shc)-mediated oxidative stress [39]. *p66Shc*^−/−^ diabetic mice were protected against endothelial dysfunction owing to less oxidative stress [39,40].

## Wnt and Vascular Smooth Muscle Cells: Proliferation, Migration and Apoptosis

The progression of atherosclerosis is associated with proliferation and migration of VSMCs, facilitated by oxidized-low density lipoproteins (ox-LDLs) [41]. VSMC proliferation is a hallmark of plaque erosion [42]. Smooth muscle cells (SMCs) are primarily localized in the medial layer of normal coronary arteries and are contractile in phenotype and have low proliferative rates. However, activation and dedifferentiation of SMCs can be seen in atherosclerosis and vessel wall injury. VSMC undergo a phenotypic switch that allows their migration to subintima, where they proliferate and deposit extracellular matrix (ECM) and contribute to atherosclerosis formation [43]. In plaque erosion the activation of platelet-derived growth factor (PDGF) in response to endothelial injury triggers VSMC proliferation.

On the other hand, VSMC may play a protective role against plaque rupture by building a fibrous cap. Intriguingly, both activation and impairment of Wnt signaling have been shown to trigger VSMC proliferation.

Increased expression of LRP6 in atherosclerotic plaques has been reported. Since Wnt induces cell proliferation and migration the increased expression of LRP6 in atherosclerotic lesion has been be linked to disease progression. However, it has been also argued that increased expression of LRP6 is a compensatory mechanism to reduce inflammation. Wnt/TCF signaling may modify SMC migration, proliferation and apoptosis during atherosclerosis process by altering ECM and MMP composition [43]. The expression of multiple ECM constituents such as fibronectin 21, versican 22 and matrix degrading metalloproteinases (MMPs) such as MMP-2, MMP-3, MMP-7, MMP-9, MMP-13, MMP-14 and MMP-26 is controlled by Wnt signaling. It has also been shown that transforming growth factor beta/SMAD3, (TGF-β/SMAD3) stimulation of canonical Wnt secretion can promote SMC proliferation via stabilization of β-catenin [44].

On the other hand, mice with the human LRP6^R611C^ loss of function mutation develop proliferative CAD caused by VSMC trans-differentiation. This effect was linked to increased Sp-1 mediated PDGF activation and reduced TCF7L2 activities [15]. The administration of rmWnt3a to *LRP6*^*R611C*^ mice normalized enhanced VSMC differentiation and reduced proliferation after carotid injury. Accordingly, heterozygous TCF7L2 mice have been shown to have excessive VSMC proliferation [45]. Studies by Towler lab has shown that VSMC-specific LRP6 disruption in LDLR^−/−^ mice results in increased vascular calcification [46].

The role of Wnt in the regulation of SMC migration is less understood. The inhibition of GSK-3β is needed for NFAT activation, which promotes arterial SMC migration during wound repair. Arterial SMC migration is promoted by JNK activation of Ca^2+^/calmodulin-dependent protein kinase (CAMKII) [47]. The apoptosis of SMCs is induced by diverse factors that include oxidative stress stimulated by reactive oxygen and nitrogen species, IL-1β, Interferon gamma (IFN-γ) and ox-LDL. A number of β-catenin target genes regulate cell survival such as IGF-1 and connective tissue growth factor/cysteine-rich 61/nephroblastoma overexpressed (CCN) growth factor family. Several other β-catenin responsive genes, including transcription factors c-jun, fra-1, Sox9 and ID2 are responsible for indirect transcriptional activation of survival genes [43]. Diminished β-catenin levels have been shown to trigger arterial SMC apoptosis via inhibition of cell cycle protein peptidyl-prolyl cis/trans isomerase (Pin1) [48]. Treatment VSMCs with Wnt5a resulted in increased β-catenin and TCF signaling whereas the treatment with H_2_O_2_ inhibited the canonical pathway [49]. In this background it was shown that the survival of arterial SMC relies upon β-catenin induction of WISP-1. Accordingly, Wnt7b^−/−^ mice display increased SMC apoptosis [43]. The exact role of β-catenin in survival of SMCs in the fibrous cap remains to be determined.

The differentiation of arterial and venous SMCs is also regulated by the Wnt pathway, which acts in conjunction with BMP pathways. Wnt3a promotes the expression of myofibroblast marker, SM-22-α. Pulmonary SMC differentiation is known to be controlled by Wnt7b via canonical Fzd1, −10 and LRP5. SIRT7, a member of a class III histone deacetylases protein family, elucidates a protective role against atherosclerosis by containing SMC proliferation and migration via Wnt/β-catenin pathways [41]. In contrast, increased DKK1, a canonical Wnt inhibitor, in carotid plaques resulted in reduced SMC proliferation, migration and survival adding to the intricacy of Wnt contribution in atherosclerosis.

Wnt signaling also plays an important role in vascular calcification, a vital process contributing to atherosclerosis. Bone formation during the embryonic stage has been found to have similarities with vascular calcification where Wnt signaling partakes a key role. Bone anabolism involves a homeodomain transcription factor, Msx2 which has been noted to upregulate Wnt3A and Wnt7A and downregulate DKK1 in primary aortic myofibroblasts [2]. Towler’s group demonstrated enhanced aortic osteochondrogenic programs and increased circulating osteopontin by SMC (SM22) -specific disruption of LRP6 in LDLR^−/−^ compared to LDLR^−/−^ mice. Loss of LRP6 in SMC promoted aortic calcification through activation of noncanonical Wnt signals [46]. A system biology approach showed that LRP6 suppresses osteogenic program by inhibiting USF1 and upregulating Jmjd6 [46].

## Wnt Signaling in Lipid Metabolism

The discovery of *LRP6*^*R611C*^ mutation in kindred with autosomal dominant early CAD, features of the metabolic syndrome, and hyperlipidemia provided the first link between impaired Wnt signaling and hyperlipidemia [25]. The screening of two hundred white Americans with early onset familial CAD and metabolic syndrome led to discovery of three other novel mutations that co-segregated with the metabolic traits in the kindreds of the affected subjects [50]. The functional characterization of one of the variants showed that it acts as a loss of function mutation and impairs Wnt signaling. Low-density lipoprotein (LDL) uptake is reduced in the splenic B cells of LRP6^+/−^ mice compared to wild-type littermates [51]. LRP6 was identified as a receptor for LDL endocytosis and clearance. LDLR internalization was also shown to be severely diminished when LRP6 was knocked down and was restored after LRP6 was reintroduced. Further analysis revealed that LRP6^WT^ forms a complex with LDLR, clathrin, and ARH and undergoes a clathrin-mediated internalization after stimulation with LDL [52]. Mice with *LRP6*^*R611C*^ mutation develop elevated plasma LDL and TG levels and fatty liver. Further investigation showed that *LRP6*^*R611C*^ mutation triggers hepatic *de novo* lipogenesis, lipid and cholesterol biosynthesis, and apoB secretion [53].

The nutrient-sensing pathway mTOR is responsible for lipid homeostasis in the liver. The study showed that Wnt activates mTOR pathway via TCF7L2 transcription of insulin-like growth factor 1 (IGF1) which results in elevated expression and activation of sterol regulatory element-binding protein (SREBP1) and *de novo* lipogenic pathway. Increased lipid synthesis and non-alcoholic hepatic steatosis is tightly associated with mTORC1 signaling activation [54]. LRP6 mutant mice (Lrp6^R11C^) had increased TG and cholesterol synthesis leading to high liver fat via IGF1-AKT-Mtor-SREBP1/2 pathway activation. Conspicuously, LRP6 mutant mice showed activation of lipogenic enzymes (acetyl CoA carboxylase), fatty acid synthase, SCD1, diglyceride acyltransferase 1 and elongation of long-chain fatty acids (ELOVL) family member 6 was seen [55]. Furthermore, plasma levels of TG, LDL and total cholesterol were significantly reduced with systemic in vivo administration of Wnt3a to LRP6-R611C homozygous mice [15].

## Wnt and Metabolic Syndrome

The metabolic syndrome (MetS), affecting nearly 40% of the adult United States population is a major risk factor for cardiovascular disease. Other constituents of MetS comprise of: hypertension, insulin resistance, dyslipidemia, a prothrombotic state and proinflammatory state. Wnt signaling has a direct link to metabolic syndrome. Human LRP6 mutations (R611C, R473Q, R360H and N433S) have been associated with MetS. LRP6 has been shown to play an important role in LDLR internalization [52]. LDLR internalization and LDL uptake has been shown to be compromised in fibroblasts of LRP6-R611C mutation carriers. LRP6-R611C mutation also increased LDL synthesis, *de novo* lipogenesis and VLDL secretion in mice. This mutation also caused bon-alcoholic fatty liver disease (NAFLD) in mice via non-canonical Wnt pathway activation and subsequent activation of TGF pathway activation. Decreased IR expression and increased mTORC1 pathway activation, resulting in enhanced IRS1 serine phosphorylation due to the loss of TCF7L2 were found to be a cause of insulin resistance in patients with the LRP6-R611C mutation [15].

Epoxyeicosatrienoic acid (EET) is a well-studied signaling molecule with vasodilatory, anti-inflammatory, increased insulin sensitivity and anti-apoptotic properties. EET is a CYP450 metabolite of arachidonic acid. EET triggers production of heme oxygenase 1 (HO-1), and suppresses the master regulator of adipogenesis, PPARγ. Furthermore, HO-1 inhibits Mesoderm specific transcript (MEST) proteins that are responsible for enlargement of adipocytes and expansion of adipose tissue. Administration of agonist of EET to mice has caused decreased adiposity and increased adiponectin levels. HO-1 is upstream of Wnt1 expression. Thus, it transactivates VEGF and erythroblast transformation-specific factor 1 (ETs1), which enhance angiogenesis and decrease angiotensin mediated myocardial fibrosis. Increased reactive oxygen species (ROS) levels are a hallmark of obesity, type 1 diabetes and impaired mitochondrial function. Mice on high fat diet show increased ROS and reduced levels of EET. However, upon introduction to EET agonist, ROS levels declines in these mice due to the action of HO-1 [56–58].

Additionally, increased HO-1 in the presence of EET, has reported in upregulation of Akt signaling, which in turn enhances eNOS production. Increased eNOS activity results in microvascular vasodilation when EET is overexpressed in mice. Similarly, enhanced EET reduce myocardial fibrosis and inflammation, and results in reduced hypertrophy and improved diastolic function [56,59].

## Wnt Contribution in Macrophage Activation

Macrophages are both a source and receiver of Wnt signals. Several Wnt molecules, including Wnt3a, Wnt4, Wnt5a, Wnt7a, Wnt7b, Wnt10a, Wnt11 and Wnt16 have been shown to be involved in macrophage biology [60,61]. Studies have shown that macrophage formation is diminished by Wnt5a and Wnt11 while it is promoted by inhibiting Wnt11 [62]. Since Wnt5a and Wnt11 as part of noncanonical Wnt pathway are known to inhibit β-catenin, it is postulated that macrophage specification requires the activation of β-catenin. Evidence suggests that Wnt signaling also regulates macrophage phagocytosis. Wnt5A signaling maintains a steady-state expression of CD14 and IFNβ, two molecules that facilitate pathogen clearance through the initiation and propagation of macrophage TLR signaling during phagocytosis and activation of immune responses [63]. Wnt5A signaling facilitates Rac1- Disheveled-lipid raft-dependent phagocytosis of bacteria and other foreign matter through modulations of the actin cytoskeleton. LRP5 appears to play a key role in macrophage phagocytosis and clearance of lipids. Accordingly, LRP5 knockout mice develop atherosclerosis with a high fat diet. LRP5 also contributes to macrophage motility. LRP5 deficient mice also showed embryonic eye hypervascularization due to failed macrophage-induced endothelial cell apoptosis [60,62]. In contrast, Wnt7a has been shown to reduce the phagocytic capacity of M-MDMs, decrease interleukin-10 (IL-10) and IL-12 secretion and increase IL-6 secretion. This is associated with reduced surface molecule expression of CD14, CD11b, CD163 and CD206, which are responsible for phagocytosis. Accordingly, monocyte derived macrophages of Wnt7a^−/−^ mice exhibited increased CD11b surface levels.

## Wnt Signal Pathways in Myocardial Infarction

Myocardial infarction (MI) is the major cause of death worldwide. The Wnt pathway has been shown to play an important role in the post-MI heart and pose as a therapeutic interventional target. MI comprises of three stages: inflammation, granulation tissue formation and fibrosis. Inflammatory cell infiltration leads to chemokine and cytokine release with further macrophage recruitment and granulation tissue formation. Angiogenesis follows with necrotic debris clearance. Collagen is produced by myofibroblasts substituting lost cardiomyocytes with fibrosis. The upregulation of Wnt2, Wnt4, Wnt10b, and Wnt11 5 days after MI followed by the upregulation of Wnt1, 1 to 14 days later and of Wnt4 in the following 7 to 14 days has been reported [11,64,65]. In a mouse model mimicking human myocardial infarction, the upregulation of Wnt2, Wnt4, Wnt10b and Wnt11 expression was seen whereas the downregulation of Wnt7B was noted. Increased expression of FZD1, FZD2, FZD5 and FZD10 and reduced expression of FZD8 was also seen [64,66]. In another study, Wnt2B, Wnt5A and to a smaller extent WNT9a were upregulated in the injured epicardial layer. In the myocardium, Wnt3A, Wnt4, Wnt5B, Wnt6, Wnt8A, Wnt9B, and Wnt10B were upregulated [67].

The Wnt secretion is also explored in the context of myocardial wound healing. Cardiac myocytes exclusively express Wnt5a which release IL-1, IL-6 and IL-8 [11]. Heart failure following myocardial infarction is prevented by blocking of Wnt3a and Wnt5a. Accordingly the Wnt inhibitor SFRP2 promotes cardiac repair after a myocardial infarction and activates mesenchymal stem cells via BMP inhibition. SFRP5 induces resistance against acute myocardial ischemia. Mice deficient of SFRP5 develop a larger infarct size, greater cardiac myocyte death and inflammation after injury. Deficiency of SFRP5 in the infarct area resulted in greater cytokine and chemokine release and increased Wnt5a-positive macrophages. The planar cell polarity pathway JNK is greatly activated in the infarcted myocardium of mice that lacked SFRP5, JNK. SFRP5 is also highly expressed in adipocytes and acts as a cardio-protective adipokine and maybe termed as a “healthy fat marker”. Its decrease will thus result in increased occurrence of myocardial infarction in obese people [32].

Fibrosis in MI is also regulated by the Wnt pathway. Aldehyde dehydrogenase-2 (ALDH2) has been shown to downregulate β-catenin (mediated via GSK3β, Wnt1 and WISP-1) and reduce fibrosis. The therapeutic effect of small molecule Wnt inhibitors such as Pyrvinium, UM206, ICG-001, Wnt −974, GNF-6231 and CGX-321 in reducing infarct size has been shown in animal models. Wnt −974 in particular terminates Wnt3 secretion and reduces post-MI fibrosis. These inhibitors are attractive drug candidates to reduce MI cardiomyocyte death, reduce fibrosis, enhance angiogenesis and encourage cardiac regeneration [11].

Recent studies conducted in murine models have shown that treatment with EET, after an ischemic cardiac episode, can reverse endothelial dysfunction and cardiac remodeling. In the presence of soluble epoxide hydrolase (sEH), EET is converted to a less potent byproduct dihydroxyeicosatrienoic acid (DHET) reducing EET activity. However, use of sEH inhibitors, that obstruct EET metabolism to DHET, improved myocardial ejection fraction and perfusion that was observed after a left anterior descending coronary artery (LAD) ligation. Treatment of mice after an infarction, with an EET agonist, significantly improved the myocardial recovery and reduced fibrosis. Similar effects were observed when LAD mice were treated with EET agonist [68,69]. However, this phenomenon was blocked once activity of HO-1 was inhibited, suggesting downstream signaling pathways like Wnt1/, β-catenin is responsible for the observed improvements of cardiac function [70].

## Conclusion

Cardiovascular disease and particularly atherosclerosis is the number one cause of death in the world. Recent scientific advances in these areas have led to the identification of genes and pathways responsible for its pathogenesis and its risk factors. The discovery of LRP6 mutation in humans provided the first evidence for the critical role of Wnt signaling in atherosclerosis and metabolic syndrome. The *in vivo* and *in vitro* characterization of LRP6 and its mutant forms provided deep insight into its novel roles in regulation of serum LDL and TG and VSMC differentiation and its impaired function as a cause of hyperlipidemia, insulin resistance and VSMC proliferation. Since then many other studies have elicited the crucial role of Wnt signaling pathways in cardiovascular physiology and diseases. The complexity of the Wnt pathway, its interactions with other pathways and formation of a complex signaling cascade provides an intriguing yet promising outlook into future therapeutic avenues. While impaired Wnt signaling has been linked to multiple metabolic disorders and atherosclerosis, it is important to bear in mind that excessive Wnt signaling may have equally deleterious effects on cardiovascular function as evident by increased cardiovascular events after the use of Wnt activator Romosozumab for the treatment of osteoporosis [71]. Future therapeutic efforts to target this pathway has to be based on precision and consider the heterogeneity of human diseases.
